# Functional connectivity–based prediction of global cognition and motor function in riluzole-naive amyotrophic lateral sclerosis patients

**DOI:** 10.1162/netn_a_00217

**Published:** 2022-02-01

**Authors:** Luqing Wei, Chris Baeken, Daihong Liu, Jiuquan Zhang, Guo-Rong Wu

**Affiliations:** School of Psychology, Jiangxi Normal University, Nanchang, China; Ghent Experimental Psychiatry Lab, Department of Head and Skin, UZ Gent/Universiteit Gent, Ghent, Belgium; Department of Psychiatry, UZ Brussel/Free University of Brussels, Brussels, Belgium; Department of Electrical Engineering, Eindhoven University of Technology, Eindhoven, Netherlands; Department of Radiology, Chongqing University Cancer Hospital, Chongqing, China; Key Laboratory of Cognition and Personality, Faculty of Psychology, Southwest University, Chongqing, China

**Keywords:** Amyotrophic lateral sclerosis, Cognitive changes, Functional connectivity, Motor severity

## Abstract

Amyotrophic lateral sclerosis (ALS) is increasingly recognized as a multisystem disorder accompanied by cognitive changes. To date, no effective therapy is available for ALS patients, partly due to disease heterogeneity and an imperfect understanding of the underlying pathophysiological processes. Reliable models that can predict cognitive and motor deficits are needed to improve symptomatic treatment and slow down disease progression. This study aimed to identify individualized functional connectivity–based predictors of cognitive and motor function in ALS by using multiple kernel learning (MKL) regression. Resting-state fMRI scanning was performed on 34 riluzole-naive ALS patients. Motor severity and global cognition were separately measured with the revised ALS functional rating scale (ALSFRS-R) and the Montreal Cognitive Assessment (MoCA). Our results showed that functional connectivity within the default mode network (DMN) as well as between the DMN and the sensorimotor network (SMN), fronto-parietal network (FPN), and salience network (SN) were predictive for MoCA scores. Additionally, the observed connectivity patterns were also predictive for the individual ALSFRS-R scores. Our findings demonstrate that cognitive and motor impairments may share common connectivity fingerprints in ALS patients. Furthermore, the identified brain connectivity signatures may serve as novel targets for effective disease-modifying therapies.

## Author Summary

Amyotrophic lateral sclerosis is recognized as a multisystem disorder, and currently no effective therapy is available for this devastating disease. Reliable models that can predict disease progression may facilitate the development of more efficient symptomatic treatment. This study used multiple kernel learning algorithm to identify a potential functional connectivity–based marker for cognitive and motor functioning in ALS. The results show that cognitive decline and motor progression could be predicted by seed-based functional connectivity from the medial prefrontal cortex/posterior cingulate cortex to the sensorimotor network, fronto-parietal network, and salience network. The identified brain connectivity signatures may serve as novel targets for effective disease-modifying therapies.

## INTRODUCTION

Amyotrophic lateral sclerosis (ALS) is a neurodegenerative disease characterized by progressive degeneration of lower motor neurons in the spinal cord and brainstem, and upper motor neurons in the motor cortex. Although the degenerative process predominantly affects the motor system, cognitive deficits have been described as well (Goldstein & Abrahams, 2013; Phukan et al., 2007). Cognitive dysfunction, as highlighted in a recent study, can adversely impact patient compliance with treatment and quality of life (Huynh et al., 2020).

Currently, no effective therapy is available for ALS patients, partly because of disease heterogeneity and an imperfect understanding of the pathophysiological processes (Kiernan et al., 2021). Further improvements in symptom management depend on advances in the understanding of the origins and progression of this devastating neurological disorder (Hardiman et al., 2011; Kiernan et al., 2021). The development of novel biomarkers to objectively assess disease progression may refine the therapeutic trial design, thereby facilitating the translation of novel therapies into the ALS clinic (Kiernan et al., 2011, 2021).

According to former research, motor and cognitive impairments in ALS are associated with pathological lesions of motor networks and a progressive spread to extramotor cortical and subcortical areas (Braak et al., 2013; Brettschneider et al., 2013). Interestingly, functional MRI (fMRI) has proven to be sensitive to the detection of inherent cerebral motor and extramotor pathology of ALS (Turner et al., 2011). In particular, resting-state fMRI (rs-fMRI) provides a new research method to explore ALS as a system failure of interconnected networks (Turner et al., 2011). For instance, ALS patients—relative to healthy controls—showed functional connectivity (FC) abnormalities in the sensorimotor network (SMN), default mode network (DMN), fronto-parietal network (FPN), and salience network (SN) (Agosta et al., 2011; Douaud et al., 2011; Mohammadi et al., 2009; Tedeschi et al., 2012; Trojsi et al., 2015). Moreover, FC changes in these networks correlated with motor severity or cognitive test scores (Agosta et al., 2011, 2013; Trojsi et al., 2015). Previous rs-fMRI studies on ALS patients primarily focused on revealing FC differences at a group-wise level or employing univariate analytical techniques to identify brain–behavior relationships. However, FC patterns are reported to be unique for individuals (Finn et al., 2015) and could be used as a predictor for clinical variables (e.g., symptom severity and treatment outcome) in neurological and psychiatric disorders (e.g., Alzheimer’s disease and major depressive disorder) (Ju et al., 2020; Lake et al., 2019; Lin et al., 2018; Yip et al., 2019). The accumulating evidence suggests that FC patterns can also serve as predictors of disease evolution of ALS at the individual level.

The aim of the current study in ALS was to identify individualized FC-based predictors for cognitive and motor function by using machine learning–based approaches. Compared to univariate analytical techniques, the machine learning models could protect against overfitting by testing brain–behavior relationships in a novel sample and provide a neuroimaging signature with high potential for clinical translation (Jiang et al., 2018; Shen et al., 2017; Yip et al., 2019). Given that previous studies on ALS patients highlighted connectivity abnormalities in the SMN, DMN, FPN, and SN (Agosta et al., 2011; Douaud et al., 2011; Mohammadi et al., 2009; Tedeschi et al., 2012; Trojsi et al., 2015), FC patterns within and between these canonical networks were chosen for the machine learning model. Especially, the core systems of the DMN, the medial prefrontal cortex (MPFC) and posterior cingulate cortex (PCC) (Andrews-Hanna, 2012; Fransson & Marrelec, 2008) were selected as regions of interest (ROIs), based on their vital roles in the pathogenesis of cognitive impairment (Anticevic et al., 2012; Binnewijzend et al., 2012; Ding et al., 2014; Pardo et al., 2007). We hypothesized that FC patterns from the MPFC and PCC to the remaining regions of the SMN, DMN, FPN, and SN would contribute to the prediction of global cognitive functioning in ALS. Additionally, since ALS-specific cognitive changes are coupled with more severe motor decline (Chiò et al., 2019; Crockford et al., 2018; Elamin et al., 2013), we also tested whether the FC model for global cognition can be extrapolated to the prediction of motor progression in ALS disease.

## MATERIALS AND METHODS

### Subjects and Cognitive Testing

Thirty-four ALS patients (21 male and 11 female) used in this study were recruited from the Department of Neurology at Southwest Hospital (Chongqing, China). Based on the revised El Escorial criteria (Brooks et al., 2000), these patients were diagnosed with sporadic probable or definite ALS. Severity of motor impairment was measured with the revised ALS functional rating scale (ALSFRS-R) (Cedarbaum et al., 1999). The range of ALSFRS-R scores was 21 to 45 in this work (10 patients at the range of 40–48, 18 patients at the range of 30—39, and 6 patients at the range of 20–29). Disease duration was defined as the duration from symptom onset to the scanning date (months), and disease progression rate was defined as (48-ALSFRS-R)/(disease duration) (Kimura et al., 2006). Exclusion criteria included: (1) the presence of other neurological or psychiatric disorders; (2) clinical diagnosis of frontotemporal dementia (Neary et al., 1998); and (3) family history of motor system diseases. None of the patients received riluzole therapy before.

The Mini-Mental State Examination (MMSE) (Folstein et al., 1975) and Montreal Cognitive Assessment (MoCA) (Nasreddine et al., 2005) were applied to evaluate global cognition in ALS Patients. Two subjects missed the MMSE and MoCA data, and 32 patients were used for the prediction of global cognitive functioning. All patients were right-handed based on measurements of the Edinburgh inventory. Study protocols were evaluated and approved by the Medical Research Ethics Committee of the Southwest Hospital. In accordance with the Helsinki Declaration, all participants provided written informed consent. The detailed demographic and clinical characteristics are presented in Table 1.

**Table 1.** Demographic and clinical data of ALS patients

Age	48.2 ± 11.5
Male/female	22/12
Education level (years)	6.9 ± 3.0
Onset (limb/bulbar/both)	26/8/0
El Escorial criteria (probable/definite)	13/21
Disease duration (month)	24.8 ± 26.7
Disease progression rate	1.15 ± 1.2
ALSFRS-R score	34.4 ± 7.0
MoCA score	25.1 ± 3.1
MMSE score	28.2 ± 2.1

*Note*. ALSFRS-R, revised ALS functional rating scale; MoCA, Montreal Cognitive Assessment; MMSE, Mini-Mental State Examination.

### Data Acquisition

Image data were acquired using a Siemens 3T Tim Trio scanner. Functional images were acquired using an echo-planar imaging sequence with the following settings: TR/TE = 2,000 ms/30 ms, flip angle = 90°, FOV = 192 × 192 mm^2^, matrix = 64 × 64, slices = 36, thickness/gap = 3 mm/1 mm, voxel size = 3 × 3 × 3 mm^3^. For each subject, a total of 240 volumes were acquired during which participants were instructed to keep their eyes closed but stay awake for a period of 480 s. High-resolution anatomical images were obtained using a magnetization-prepared rapid gradient-echo (MP-RAGE) sequence with the following settings: TR/TE = 1,900 ms/2.52 ms, flip angle = 9°, matrix size = 256 × 256, slices = 176, thickness = 1 mm, and voxel size = 1 × 1 × 1 mm^3^.

### Data Preprocessing

Functional images preprocessing was performed using fMRIPrep (version 1.4.1) (Esteban et al., 2019). Briefly, the T1-weighted (T1w) image was corrected for intensity nonuniformity with N4BiasFieldCorrection (ANTs) and used as T1w-reference throughout the workflow. Then, a blood oxygen level–dependent (BOLD) reference volume and its skull-stripped version were generated using a custom methodology of fMRIprep. The BOLD reference was coregistered with the T1w reference (bbregister, FreeSurfer). Coregistration was configured with 9 degrees of freedom to account for the distortions remaining in the BOLD reference. Head-motion parameters with respect to the BOLD reference are estimated before any spatiotemporal filtering using mcflirt (FSL). After that, the BOLD runs were slice-time corrected using 3dTshift (AFNI) and resampled into the MNI152NLin2009cAsym standard volumetric space. Framewise displacement (FD) (Power et al., 2012) was calculated for each functional run.

To remove the confounds due to physical and physiological noise, the following nuisance regressors were simultaneously included in a linear regression model: six realignment parameters and their temporal derivatives (Power et al., 2012), physiological noise estimated using the anatomical component correction method (aCompCor, the top five principal components from the union of cerebrospinal fluid and white matter masks calculated in T1w space) (Behzadi et al., 2007), and first-order Legendre polynomial. Finally, temporal band-pass filtering (0.01 ∼ 0.1 Hz) was applied to the residual time series. For further details of the pipeline, please see the section corresponding to the workflows in fMRIPrep’s documentation (https://fmriprep.org/en/latest/workflows.html).

### Regions of Interest–Based Functional Connectivity Analyses

**Figure 1.**
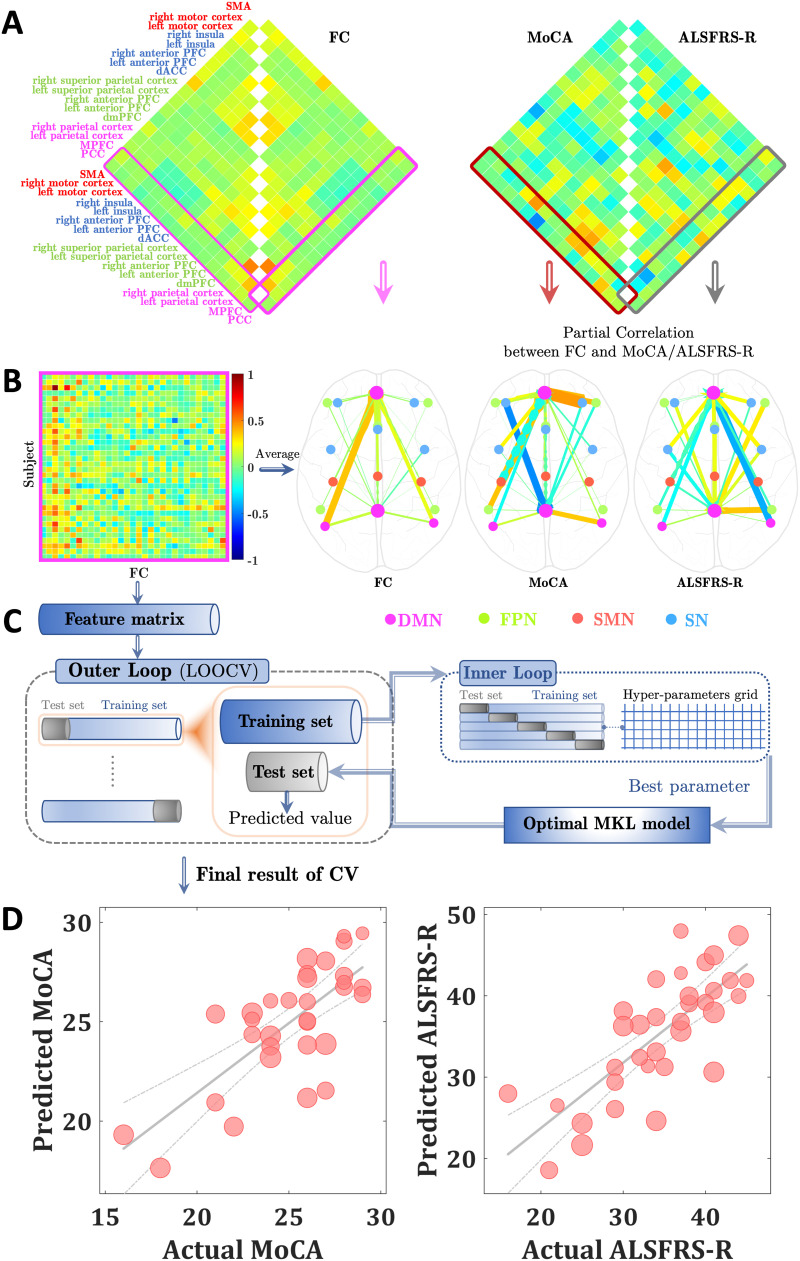
(A) Functional connectivity maps (left panel) and their relationships (right panel) with Montreal Cognitive Assessment (MoCA) (left part matrix) and amyotrophic lateral sclerosis functional rating scale (ALSFRS-R) scores (right part matrix), obtained by partial correlation analysis (with age, gender, and mean framewise displacement as covariates). (B) Functional connectivity (FC) features for the prediction of MoCA and ALSFRS-R scores. (C) Flow chart of the nested cross-validation. (D) Scatter plot showing actual and predicted MoCA (*r* = 0.737, *p* = 0.015) / ALSFRS-R (*r* = 0.764, *p* = 0.003) scores. The size of the scatter point is proportional to age.

On the basis of former research (Raichle, 2011), the supplementary motor area (SMA) and bilateral motor cortex constituted the SMN, the MPFC, PCC, and bilateral parietal cortex constituted the DMN, and the dorsal medial prefrontal cortex (PFC), bilateral anterior PFC, and superior parietal cortex constituted the FPN. For the SN, it comprised five key nodes (i.e., dorsal anterior cingulate cortex [dACC], bilateral anterior PFC and insula) as described in Menon (2015). Especially, the MPFC and PCC were chosen as two seed regions based on their crucial role in cognitive decline. All above-mentioned brain regions and corresponding coordinates (Table 2) used in this work were obtained from Raichle (2011). For each individual subject, we computed the Pearson correlation coefficient between the averaged time course of each seed region (sphere with radius = 5 mm) and the time courses of the remaining network’s nodes. Then the generated Pearson’s *r* maps were converted to *z* maps using Fisher’s *z* transformation (Figure 1A and 1B). The above FC analyses were performed using the rsHRF toolbox (Wu et al., 2021) (https://www.nitrc.org/projects/rshrf).

**Table 2.** Brain regions and corresponding coordinates applied for FC analyses

Network/region	MNI coordinates
**SMN**	
SMA	0, −21, 48
Left motor cortex	−39, −26, 51
Right motor cortex	38, −26, 48
**DMN**	
MPFC	−1, 54, 27
PCC	0, −52, 27
Left parietal cortex	−46, −66, 30
Right parietal cortex	49, −63, 33
**FPN**	
dmPFC	0, 24, 46
Left anterior PFC	−44, 45, 0
Right anterior PFC	44, 45, 0
Left superior parietal cortex	−50, −51, 45
Right superior parietal cortex	50, −51, 45
**SN**	
dACC	0, 21, 36
Left anterior PFC	−35, 45, 30
Right anterior PFC	32, 45, 30
Left insula	−41, 3, 6
Right insula	41, 3, 6

*Note*. SMA, supplementary motor area; MPFC, medial prefrontal cortex; PCC, posterior cingulate cortex; dmPFC, dorsal medial prefrontal cortex; PFC, prefrontal cortex; dACC, dorsal anterior cingulate cortex.

### Individualized Prediction

We employed a multiple kernel learning (MKL) with lasso regularization algorithm to predict cognitive (MMSE and MoCA) and motor (ALSFRS-R) test scores, implemented in the PRoNTo (https://www.mlnl.cs.ucl.ac.uk/pronto, version 2.1.1) and SpicyMKL toolbox (https://ibis.t.u-tokyo.ac.jp/suzuki/software/SpicyMKL/, version 3) (Suzuki & Tomioka, 2011). A nested cross-validation was used simultaneously for the selection of the hyperparameter (5-fold cross-validation for the inner loop to optimize the model’s hyperparameter, that is, grid search soft-margin C: [0.0001, 0.001, 0.01, 0.1, 1, 10, 100], then the best C value was used for the outer loop) and assessment of generalization capacity (leave-one-out for the outer loop, see Figure 1C). Age, gender, and mean FD were combined in one single linear kernel. For each FC, a linear kernel was computed. All kernels were mean centered and normalized before MKL modeling. The Pearson correlation coefficient (*r*) and root mean squared error (rMSE) were calculated between the actual and predicted motor and cognitive test scores for the overall predictive performance. Permutation tests were carried out to assess the statistical significance of the correlation coefficient and rMSE (randomly shuffled cognitive and motor scores 1,000 times). The results were considered significant if the *p* value < 0.05/3 = 0.017 (Bonferroni correction to account for multiple comparisons).

To examine the specificity of the SMN, DMN, FPN, and SN in predicting cognitive and motor test scores, the visual (left visual system: −7, 83, 2; right visual system: 7, 83, 2) and auditory networks (left auditory system: −62, −30, 12; right auditory system: 59, −27, 15) that are not directly related to ALS disease pathology were also included in the machine learning model (Raichle, 2011). The predictive power of the model with and without the two networks was compared using Meng’s Z test (Meng et al., 1992).

Besides, to further clarify the specificity of FC from the seed regions to the FPN or SN or SMN in the prediction of individual cognitive and motor scores, a computational lesion analysis was further performed (Feng et al., 2018; Wang et al., 2021). Concretely, the FC patterns excluding connectivity from seed regions to the FPN or SN or SMN were employed for the prediction of MoCA and ALSFRS-R scores. The predictive power for lesion and no lesion model was compared using Meng’s Z test.

## RESULTS

The MKL regression model showed that FC from the MPFC and PCC to the DMN regions (i.e., bilateral parietal cortex) as well as to the SMN, FPN, and SN regions were predictive for the individual MoCA and ALSFRS-R scores. As shown in Figure 1D, the predicted MoCA and ALSFRS-R scores significantly correlated with the actual MoCA (*r* = 0.737, *p* = 0.015; rMSE = 2.172, *p* = 0.014) and ALSFRS-R (*r* = 0.764, *p* = 0.003; rMSE = 5.006, *p* = 0.006) scores. No prediction was found for the MMSE scores (*r* = 0.013, *p* = 0.573; rMSE = 5.746, *p* = 0.438).

After inclusion of the visual and auditory networks in the MKL model, FC patterns were not predictive for the MoCA (*r* = 0.369, *p* = 0.123; rMSE = 3.307, *p* = 0.086), MMSE (*r* = 0.203, *p* = 0.291; rMSE = 3.726, *p* = 0.442), and ALSFRS-R scores (*r* = 0.309, *p* = 0.157; rMSE = 9.057, *p* = 0.160). Moreover, the predictive power of the model for MoCA and ALSFRS-R scores decreased dramatically (MoCA: Meng’s Z = 2.637, *p* = 0.004; ALSFRS-R: Meng’s Z = 3.317, *p* < 0.001). These results may indicate that the SMN, DMN, FPN, and SN are specific to the individualized prediction of cognitive and motor functions in ALS patients.

The computational lesion analysis revealed that FC patterns excluding connectivity with the SMN were not predictive for ALSFRS-R (*r* = 0.152, *p* = 0.286; rMSE = 15.063, *p* = 0.226; Meng’s Z = −3.523, *p* < 0.001) and MoCA (*r* = 0.187, *p* = 0.295; rMSE = 6.756, *p* = 0.168; Meng’s Z = −3.740, *p* < 0.001) scores. After exclusion of connectivity to the FPN, FC patterns were not predictive for ALSFRS-R scores (*r* = −0.267, *p* = 0.881; rMSE = 15.946, *p* = 0.736). Additionally, FC after lesion of the connectivity with SN was not predictive for MoCA scores (*r* = −0.015, *p* = 0.553; rMSE = 8.631, *p* = 0.842). More details about the lesion results are presented in Table 3.

**Table 3.** Results of prediction with specific lesion analysis

Lesion	Dependent variable	Predictive power				Lesion vs. no lesion model	
		rMSE	*p* value^a^	*r*	*p* value^a^	Meng’s *Z*	*p* value^b^
Seeds-SMN	MoCA	6.756	0.168	0.187	0.295	−3.740	<0.001
	ALSFRS-R	15.063	0.226	0.152	0.286	−3.523	<0.001
Seeds-FPN	MoCA	2.170	0.002	0.783	0.002	0.576	0.718
	ALSFRS-R	15.946	0.736	−0.267	0.881	−4.729	<0.001
Seeds-SN	MoCA	8.631	0.842	−0.015	0.553	−3.849	<0.001
	ALSFRS-R	8.788	0.036	0.503	0.017	−2.322	0.010

^a^ Permutation test.

^b^ Parametric test; seeds: MPFC&PCC.

## DISCUSSION

This is the first brain imaging study applying the FC-based machine learning algorithm to predict cognitive and motor function in ALS disease at single-subject level. The MKL model has identified FC within the DMN as well as between the DMN and the SMN, FPN, and SN, contributing to the prediction of global cognitive functioning in ALS. In addition, the observed FC patterns also predicted individual motor impairment scores. The current findings show that individual differences in baseline connectivity within and between large-scale neural networks contribute to variability in global cognition and motor progression in ALS disease. Furthermore, the identified predication models may provide novel biomarkers for clinical trial designs, holding the promise of the development of effective therapies for ALS patients.

Individual differences in MoCA scores are closely linked to connectivity from the MPFC and PCC to the bilateral parietal cortex, demonstrating that the DMN intranetwork connectivity was predictive for global cognitive function in ALS. The DMN, characterized by deactivation during goal-directed cognitive tasks and increased activity in self-referential processing (Buckner et al., 2008; Raichle et al., 2001), plays an important role in the pathogenesis of cognitive impairment (Binnewijzend et al., 2012; Greicius et al., 2004; Sorg et al., 2007). In particular, the MPFC and PCC were key structures for cognitive decline, typically found in normal aging (Pardo et al., 2007), as well as in mild cognitive impairments, and in Alzheimer’s disease (AD) (Binnewijzend et al., 2012). Previous studies have linked impaired connectivity within the DMN to ALS patients without dementia (Agosta et al., 2013; Mohammadi et al., 2009; Trojsi et al., 2015). Moreover, connectivity abnormalities in the DMN correlated with cognitive performance scores in ALS (Agosta et al., 2013). The current results further lend support to the assumption that ALS can be characterized by the alteration of functional networks associated with global cognition, even before the occurrence of overt dementia. As discussed above, the DMN frequently underpins the development of cognitive deficits in ALS disease. However, clinical disability scores (ALSFRS-R) could also be reliably predicted by DMN connectivity. As the correlation between DMN connectivity and motor severity has been observed in patients with ALS (Agosta et al., 2013), our results extended previous findings by showing that DMN connectivity was predictive for motor progression in newly diagnosed drug-naive individuals. According to former ALS research, cognitive impairments are associated with a more rapid decline of motor function (Chiò et al., 2019; Crockford et al., 2018; Elamin et al., 2013). This could indicate that neural networks accounting for cognitive functioning may predict motor progression in ALS. Overall, the current findings imply that the pathological process of ALS involves DMN regions, and lesions of the DMN can be predictive for the level of cognitive and motor decline.

FC between the DMN and SMN, FPN, and SN were predictive for MoCA and ALSFRS-R scores in ALS, providing further evidence for the involvement of the SMN, FPN, and SN in ALS. The SMN, FPN, and SN have been described in previous rs-fMRI studies on ALS (Agosta et al., 2011, 2013; Mohammadi et al., 2009; Tedeschi et al., 2012; Trojsi et al., 2015). In particular, the SMN is responsible for motor function, and abnormal connectivity in this network contributes to motor dysfunction in patients with ALS (Agosta et al., 2011, 2013; Mohammadi et al., 2009; Tedeschi et al., 2012; Trojsi et al., 2015). The FPN subserves attention, executive processing, planning, and working memory (Corbetta & Shulman, 2002). Altered FPN connectivity could explain the executive deficits frequently observed in ALS (Agosta et al., 2013; Tedeschi et al., 2012; Trojsi et al., 2015). The SN has been conceptualized as a bottom-up processor of salient experiences, and is involved in the initiation of cognitive control by influencing activation of the FPN and the DMN (Menon, 2011; Menon & Uddin, 2010; Seeley et al., 2007). Abnormal connectivity in the SN accounts for behavioral disturbances (e.g., apathy, irritability, aggression, disinhibition, and distractibility) in ALS patients (Phukan et al., 2007; Trojsi et al., 2015). Taken together, our current findings suggest that motor and extramotor networks are involved in ALS, supporting the notion of ALS as a multinetwork disorder.

Of note, this study showed that FC between the DMN and SMN as well as between the DMN, FPN, and SN were predictive for MoCA and ALSFRS-R scores, indicating that baseline internetwork connectivity can be used to explain individual differences in global cognitive and motor function in ALS patients. As described above about the role of the DMN and SMN, it is not surprising that global cognition and motor progression can be predicted by using the connectivity between the DMN and SMN. Interestingly, after the lesion of this internetwork connectivity, the predictive power of the model for MoCA and ALSFRS-R scores decreased significantly (i.e., FC patterns were not predictive for these two measurements). This result may demonstrate that the DMN-SMN connectivity is specific to the individualized prediction of cognitive and motor functioning in ALS.

Regarding the DMN, FPN, and SN, a triple network model proposes that functional interactions among the three core neurocognitive networks are crucial for attention control, working memory, decision-making, and other higher level cognitive functions (Chen et al., 2013; Menon, 2011). Dysfunctional couplings between the three networks may underlie the progression of cognitive deficits in Parkinson’s disease (Putcha et al., 2016). We found that functional couplings between the DMN, FPN, and SN were predictive for global cognition in ALS patients, further supporting the triple network model as a common neuronal substrate of cognitive functioning. Besides, these couplings were also predictive for individual motor deficits, suggesting that neural circuits underlying cognitive function may predict motor symptoms in ALS.

Although the MoCA scores can be predicted by interactions between the DMN, FPN, and SN, the computational lesion results have shown the specificity of the DMN-SN coupling in predicting global cognition of ALS. To support complex and flexible cognitive processes, the SN signals the DMN to reduce its activity when a salient event is detected (Menon & Uddin, 2010; Sridharan et al., 2008). Communications between the SN and DMN are crucial for efficient cognitive control (Bonnelle et al., 2012; Ham et al., 2013; Jilka et al., 2014). Moreover, successful cognition in elderly people relies on healthy coupling between the SN and DMN (Putcha et al., 2016; Tsvetanov et al., 2016), and the abnormality of this internetwork connectivity is associated with cognitive decline in AD patients (He et al., 2014). The above-mentioned findings indicate that the DMN-SN coupling is important for the maintenance of cognitive capacities. Therefore, FC patterns after a lesion of this connectivity were not predictive for global cognition in ALS. Nonetheless, the DMN-FPN coupling did not show resembling specificity in predicting MoCA scores. The cooperation between the DMN and FPN has been shown to control executive functions such as cognitive flexibility, attention, and working memory (Cole et al., 2012; Dajani & Uddin, 2015; Douw et al., 2016). The specificity of the DMN-FPN connectivity in predicting cognitive function of ALS should be further validated by using more specific cognitive measures (e.g., verbal fluency, attention, set shifting, and episodic memory) (Chiò et al., 2019; Phukan et al., 2007), since the MoCA is a general and brief cognitive screening instrument. Finally, the specificity was observed for the DMN-FPN coupling in predicting ALSFRS-R scores, implying that neural networks accounting for cognitive changes may be used to predict motor decline. A possible explanation for this finding could be that cognitive changes are coupled with greater motor decline in ALS (Chiò et al., 2019; Crockford et al., 2018; Elamin et al., 2013).

It should be pointed out that our prediction model was not validated in another independent dataset. However, the results with the holdout method indicated that FC within the DMN as well as between the DMN and the SMN, FPN, and SN could reliably predict global cognition and motor function in ALS patients (Supporting Information Figure S1, unlike the leave-one-out cross-validation, the test set is never used as the training set and vice versa). In addition, there is a lack of longitudinal data addressing the predictive model of progression rate on MoCA and ALSFRS-R scores. Notwithstanding, here we applied the disease progression rate—defined as (48-ALSFRS-R)/(disease duration)—(Kimura et al., 2006) for MKL modeling. The results showed that disease progression rate could not be predicted by the observed FC patterns (*r* = 0.403, *p* = 0.085; rMSE = 1.687, *p* = 0.074), suggesting that the FC model of global cognition cannot be extrapolated to the prediction of this measurement. More suitable FC models are needed to further verify the current finding. Furthermore, future studies with longitudinal datasets would be necessary to further predict cognitive and motor decline in ALS patients. Finally, this work only assessed global cognitive function in ALS patients. To further investigate FC-based predictive models for specific cognitive impairments, a comprehensive battery of neuropsychological tests encompassing language, memory, executive function, social cognition, and visuospatial function should be included in a future study (Chiò et al., 2019; Consonni et al., 2018; Crockford et al., 2018; Lule et al., 2018).

## CONCLUSIONS

In summary, our results show that individual differences in FC within and between large-scale neural networks contribute to variability in global cognition and motor progression, supporting the notion of ALS as a multinetwork disorder. Moreover, the identified brain connectivity signatures may provide novel biomarkers for effective therapy of ALS patients. Replication and validation using other and larger datasets are needed before these models can be confidentially used in clinical practice.

## SUPPORTING INFORMATION

Supporting information for this article is available at https://doi.org/10.1162/netn_a_00217.

## AUTHOR CONTRIBUTIONS

Luqing Wei: Methodology; Writing – original draft; Writing – review & editing. Chris Baeken: Validation; Writing – review & editing. Daihong Liu: Data curation; Investigation. Jiuquan Zhang: Conceptualization; Project administration; Supervision. Guo-Rong Wu: Methodology; Writing – original draft; Writing – review & editing.

## FUNDING INFORMATION

Luqing Wei, National Natural Science Foundation of China (https://dx.doi.org/10.13039/501100001809), Award ID: 31900764. Guo-Rong Wu, National Natural Science Foundation of China (https://dx.doi.org/10.13039/501100001809), Award ID: 61876156. Jiuquan Zhang, National Natural Science Foundation of China (https://dx.doi.org/10.13039/501100001809), Award ID: 82071883. Chris Baeken, Research Foundation - Flanders (FWO), Award ID: T000720N.

This work was also supported by the Queen Elisabeth Medical Foundation for Neurosciences, by the Ghent University Multidisciplinary Research Partnership “The Integrative Neuroscience of Behavioral Control,” a grant BOF16/GOA/017 for a Concerted Research Action of Ghent University, and by an Applied Biomedical (TBM) grant of the Agency for Innovation through Science and Technology (IWT), part of the Research Foundation - Flanders (FWO) PrevenD Project 2.0 (T000720N) and FWO project G011018N.

## TECHNICAL TERMS

ALSFRS-R: A scale measured the severity of motor impairment.
Riluzole: A medication is used to treat ALS, which may delay disease progression and prolong life.
MoCA: A scale applied to evaluate global cognitive function.
Nested cross-validation: Training model hyperparameters and evaluating model performance.
Meng’s Z test: Comparison of the correlated correlation coefficients.
Computational lesion analysis: Examining the specificity of connectivity for predictions.
