# Supplementary material for: Functional connectivity–based prediction of global cognition and motor function in riluzole-naive amyotrophic lateral sclerosis patients
Source: Netw Neurosci. 2022 Feb 1;6(1):161–74. doi: 10.1162/netn_a_00217 (PMC8959121; doi:10.1162/netn_a_00217)
Supplement: Supplementary file 1 [file netn-06-161-s001.pdf]

Wei, L., Baeken, C., Daihong, L., Zhang, J., & Wu, Guo-Wu. (2022). Supporting information for "Functional connectivity-based prediction of global cognition and motor function in riluzole-naive amyotrophic lateral sclerosis patients." *Network Neuroscience*, *6*(1), 161–174. https://doi.org/10.1162/netn_a_00217

Supporting materials

Hold-out validation

To avoid bias caused by random division, we sorted subjects according to their MoCA/ALSFRS-R scores and then assigned them into two subsets: (1st, 3rd,…), (2nd, 4th,…) (Cui et al. 2017). We take the first subset as training data and the second subset as the test dataset. Apart from the cross-validation, other prediction procedures are the same as Section 2.5.

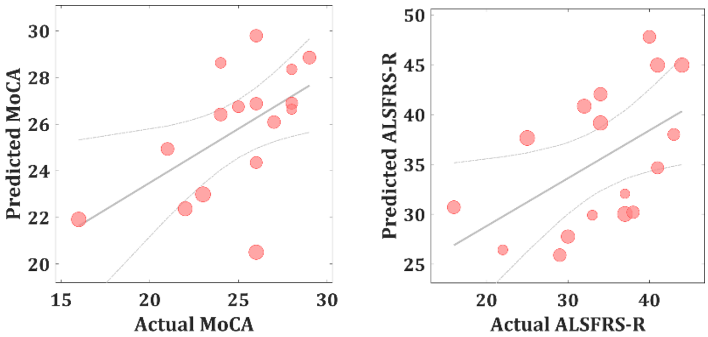

Figure S1. Scatter plot showing actual and predicted MoCA (r = 0.564, p = 0.013) / ALSFRS-R (r = 0.526, p = 0.017) scores. The size of scatter point is proportional to age.

Cui Z, Su M, Li L, Shu H, Gong G (2017) Individualized prediction of reading comprehension ability using gray matter volume. Cereb Cortex 28(5):1–17
